# Effect of timing of mother’s death on child survival in a rural HIV hyper-endemic South African population

**DOI:** 10.1186/s12889-018-6152-8

**Published:** 2018-11-06

**Authors:** Boikhutso Tlou, Benn Sartorius, Frank Tanser

**Affiliations:** 1 Discipline of Public Health Medicine, School of Nursing and Public Health, University of KwaZulu-Natal, Durban, South Africa; 2 Africa Centre for Health and Population Studies, University of KwaZulu-Natal, Mtubatuba, South Africa; 3 Centre for the AIDS Programme of Research in South Africa –CAPRISA, University of KwaZulu-Natal, Durban, South Africa; 4 Research Department of Infection & Population Health, University College London, London, UK

**Keywords:** Timing, Maternal mortality, Child survival, Socio-economic status, HIV, Rural South Africa

## Abstract

**Background:**

Maternal mortality remains a tragedy and a key determinant for child survival. There is increasing evidence that the hazard ratio of demising for young children escalates after the death of their mothers, but few studies has been done in rural areas were HIV/AIDS is more prevalent. The aim of this study is to investigate the survival of children who lost their mothers soon or after their births in a rural setup with high HIV prevalence in South Africa.

**Methods:**

This study used a data set from Africa Health Research Institute in rural South Africa.The study population comprised children (0–10 years of age) from 2000 to 2014. We employed a Cox regression modelling approach to estimate greatest temporal hazard of the child after the death of their mothers, accounting for the confounding influence of wealth index of the household and HIV status of the mother.

**Results:**

We found 62,600 live births, and that 2191 children died when they were less than or equal to 10 years old. The mortality rates for < 5 and 5–9 years is 882.25 and 117.75 per 1000 live births respectively, with a maternal mortality rate of 447.3 deaths per 100,000 live births from 2000 to 2004. Child mortality risk was very high in less than 6 weeks after their mother’s death (HR 3.45 [95%CI: 1.3–6.54]), and decreased drastically after 3 years following her death (HR 0.8 [0.2–6.3]). This increased risk was more pronounced among children aged less than 1 month and living in poor households.

**Conclusions:**

Children (less than 10 years) in rural households are at their highest risk of dying within 6 weeks of mother’s death and this risk decreases substantially after the highly vulnerable window. This indicates that the period of mother’s death does play a critical role on the survival of her children.Thus, understanding this risk and its timing in relation to a mother’s death is critical to guide interventions and stress the relevance of assessing the interaction between clinical care and socio-economic programs in addressing the needs of orphans.

## Background

One of the leading causes of death among adult women globally is maternal mortality and approximately 830 women die from pregnancy- or childbirth-linked complexities around the world daily [1].Maternal mortality is characterized by extensive disparities within and across countries. For example; maternal mortality ratios in poverty-stricken countries are approximately fifteen times more than those in affluent nations, and across developing countries, the poverty-stricken women are at a considerable risk of demise at the time of gestation or childbirth [2, 3]. The main direct and indirect causes of maternal mortality are obstetrical, selected infectious or non-communicable causes believed to be strengthened by pregnancy.

In addition to that, the emergence of HIV/AIDS particularly in third world countries has exacerbated maternal mortality for most women in their reproductive ages [4].In most developing countries when a woman dies, the children are often exposed to immense risks of poverty and mortality due to neglect.

Research has shown that in third world countries, the survival chances of a child are limited towards their mothers’ death [5, 6]. Previous studies done in the Africa Health Research Institute have indicated that maternal deaths and child survival are highly correlated [7–9]. Past studies done in other typical rural areas (South Africa [6], Bangladesh [10], Benin [11], Haiti [12] and Kenya [13]) have indicated that a mother’s death significantly reduces the survival chances of her children, particularly in the early stages of life even though the impact significantly decreases with an increase on age.

The HIV prevalence in sub-Saharan Africa has also exacerbated, the already critical dilemma over the past twenty years [14–16]. As already, alluded, maternal mortality is also explicitly related to child mortality due to obstetric complications [17]. A motherless infant has a high probability of mortality due to malnutrition and an increased susceptibility to infections [18]. The space-time associations between child and maternal mortality are crucial in guiding policy implementations and interventions more especially in communities and areas more susceptible to female adult mortality. A woman’s environmental health status, education and socio-economic status play a critical act on the survival of her children [19]. Further evidence is urgently required to help guide interventions in third world countries particularly those were HIV prevalence rate is still high.

Study results differ depending on household socio-economic indices, given inequalities on household organization and resources.Socio-economic status has a positive effect on both death and birth rates, normally a high socio-economic index reduces birth and death rates [20]. An increase in electricity, clean water, income, housing conditions, land ownership, education and sanitation improves the health status of a given community [21]. In addition, healthy populations positively influences economic growth and this positively reduces poverty in any population.

This study aims to assess the association the risk of young children dying with the timing of the deaths of their mothers, and particularly to investigate critical periods where the risk was high after their mothers’ deaths in poor rural settings. This will add to the emerging evidence by investigating the impact of mother death on child mortality in rural sub-Saharan Africa after adjusting key confounding influences such as household socio-economic index and women’s HIV status.

## Methods

### Study setting and data collection

**Fig. 1:**
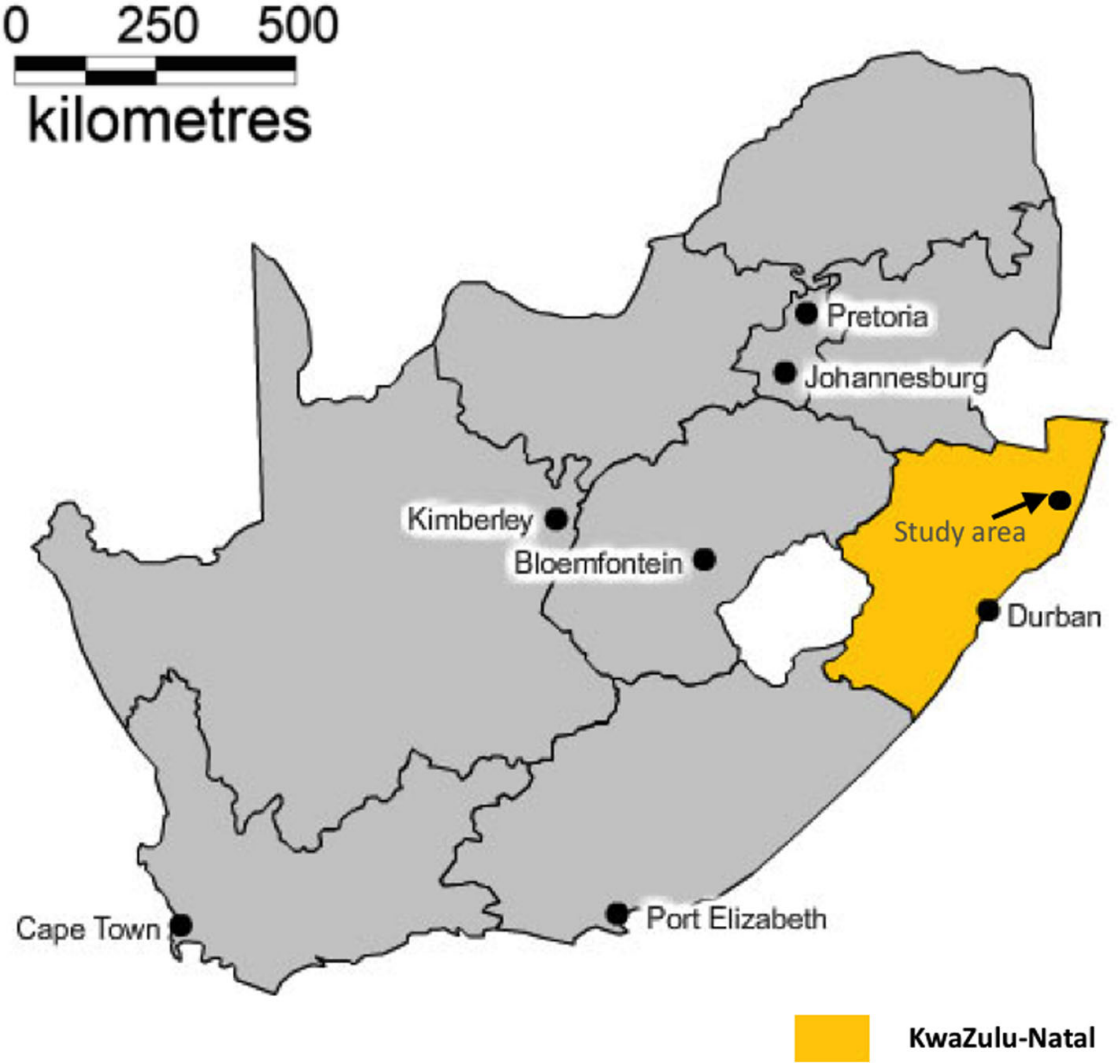
Location of the Africa Centre’s study area in KwaZulu-Natal Province, South Africa [42]

The Africa Health Research Institute (Fig. 1) is situated closer to the Mtubatuba market in Northern KwaZulu-Natal. The study area is 438 km^2^ and comprises approximately 11,000 households with an estimated population of 85,000 people who speak isiZulu as it has been already been described in detail in previous studies [22–24] . Each household has an average size of 7.9 members. Most households rely on governmental grants and pensions as a source of income rather than agriculture as one would expect in a rural area. Follow up on individuals who are residents of the study area is done twice a year and the data collected is mainly demographic, socio-economic and behavioral. All deaths recorded in the Africa Health Research Institute study area are validated through verbal autopsy interviews and this has already been described in detail previously [22, 23, 25]. In addition, the demographic and socio-economic attributes of the study participants have been extensively described in detail in previous studies [24, 26, 27].The ethical approval of this study was obtained from the Biomedical Research Ethics Committee (BREC) of the University of KwaZulu-Natal (BE 169/15).

### Variable definition

We used a subset of the full Africa Health Research Institute database for this analysis i.e. restricted to children younger than 10 years born to women during the period (2000–2014). We defined maternal death as “the death of a woman while pregnant or within 42 days of termination of pregnancy, irrespective of the duration and site of the pregnancy, from any cause related to or aggravated by the pregnancy or its management but not from accidental or incidental causes [28].” We also defined the “index child” “as the reference birth for counting the 42 days maternal death window, and non-index children were all births prior to the one affiliated with the maternal death [5].”Mother death was defined as the death of any woman of childbearing age when the child was less than 10 years. In addition, we defined the hazard ratio as the ratio of the risk of death in one group divided by the same risk in another group occurring at a given interval of time. Survival time was calculated using the number of days from the birth of children until the end of the event, which could be death/out-migration or even the censoring. We divided timing of woman death into the following categories: < 6 weeks, 6 weeks< 18 months, 18 months < 3 years, 3 years < 8 years, and ≥ 8 yearss. Our analysis classified socio economic status into three wealth indices using principal component analysis (PCA) weights of household assets [29], namely poor, medium and rich. We found 62,600 children less than 10 years born during the period 2000–2014.

### Statistical methods

We created Kaplan-Meier survival curves to graphically depict differences in survival by key explanatory variables, namely: mother death status and timing of mother death. The Kaplan-Meier analysis enables computation of survival over the given time period, even when participants are lost or followed for contrasting durations. We then calculated the survival probability by divided the number of surviving participants by the number of those at risk. However, those respondents who were lost to follow –up, demised or haven’t reached the time to an event were not treated as at risk and those lost to follow-up were regarded “censored” and were not included in the denominator. We used the log-rank test to estimate the significance of the difference between the survival curves of the levels of different variables. We assessed the proportional hazards assumption using the supremum test for proportional hazards assumption. We employed the Cox regression to model the influence of timing of mother death on child death and adjusted hazard ratios (and 95% confidence intervals) for influential predictors: wealth index, mother’s HIV status and child’s sex. We conducted all analyses using SAS 9.4(SAS Institute Inc. 2015) [30].

## Results

The demographic profile and characteristics of study participants during the study period(2000–2014) are presented in Table 1.Out of 62,600 children, an overall of 2191 children (3.5% of the sample) less than 10 years died and 280 women died when the child was younger than or 10 years old. The mortality rates for less than 5 and 5–9 years is 882.25 and 117.75 per 1000 live births respectively and a maternal mortality rate of 447.3 per 100,000 live births. The proportion of “index child” deaths (84%) was significantly higher (*p* < 0.001) than the proportion of deaths amongst the elder siblings of the “index child” (11%). Also, 36 children who died after their mother death, 23 of those deaths were HIV/AIDS and TB related. The mean age for women deaths was approximately 31 years. The major causes of deaths in the peak period between mothers and children were TB and HIV.

**Table 1:** Characteristics of children and their mothers for the periods 2000–2014, Africa Centre for Demographic Information System, South Africa

	Total children (*n* = 62,420)	Maternal deaths (*n* = 280)	Non Maternal deaths (*n* = 62,140)
*Children*			
Child Sex			
Male (%)	31,370(50.3)	140 (50)	31,230(50.3)
Female (%)	31,050(49.7)	140 (50)	30,910(49.7)
Age: years (Std. Dev.)	7.57 (2.74)	7.14 (3.03)	7.58 (2.74)
Number of Child Deaths (%)	2191(0.04)	36(0.13)	2155(0.04)
HIV/AIDS and TB-Related Child Deaths (%)	749(0.01)	23(0.08)	726(0.01)
Mean Age at Death: months (Std. Dev.)	21.71 (27.78)	24.11 (27.78)	21.67 (28.28)
Child deaths by period			
2000–2006	1516	30	1486
2007–2014	675	6	669
*Mothers who died during the period*			
Mean Age by period			
2000–2006	34.27 (8.30)		
2007–2014	35.88 (8.61)		
HIV/AIDS and TB-Related Deaths (%)		23(8.2%)	

For those mothers who died, mean age represents the average age at time of death

**Fig. 2:**
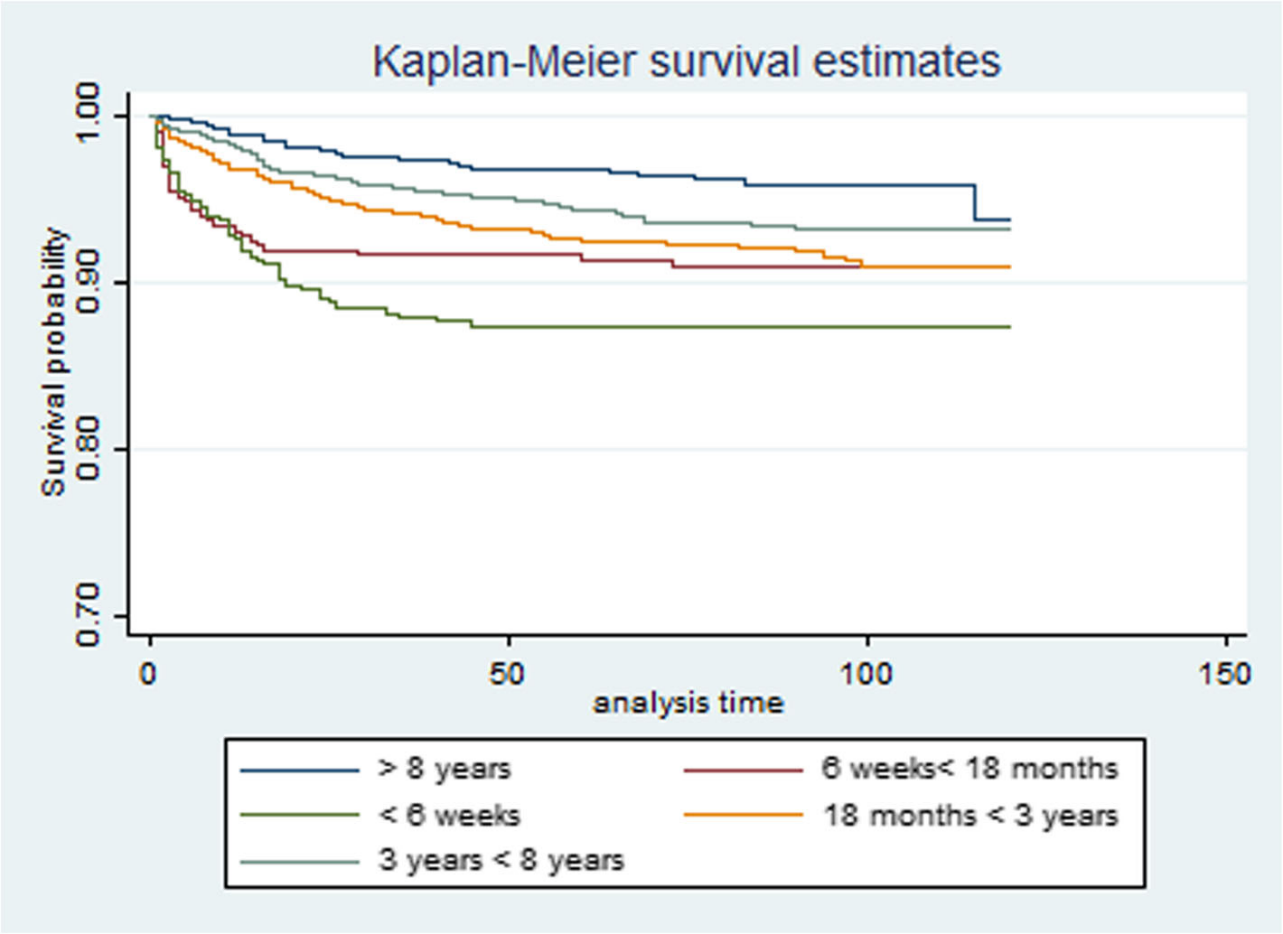
Kaplan–Meier survival curve by timing of mother death, women who die within 10 years of childbirth. Note: Log rank test for equality of survival functions: *p*-value< 0.001

**Fig. 3:**
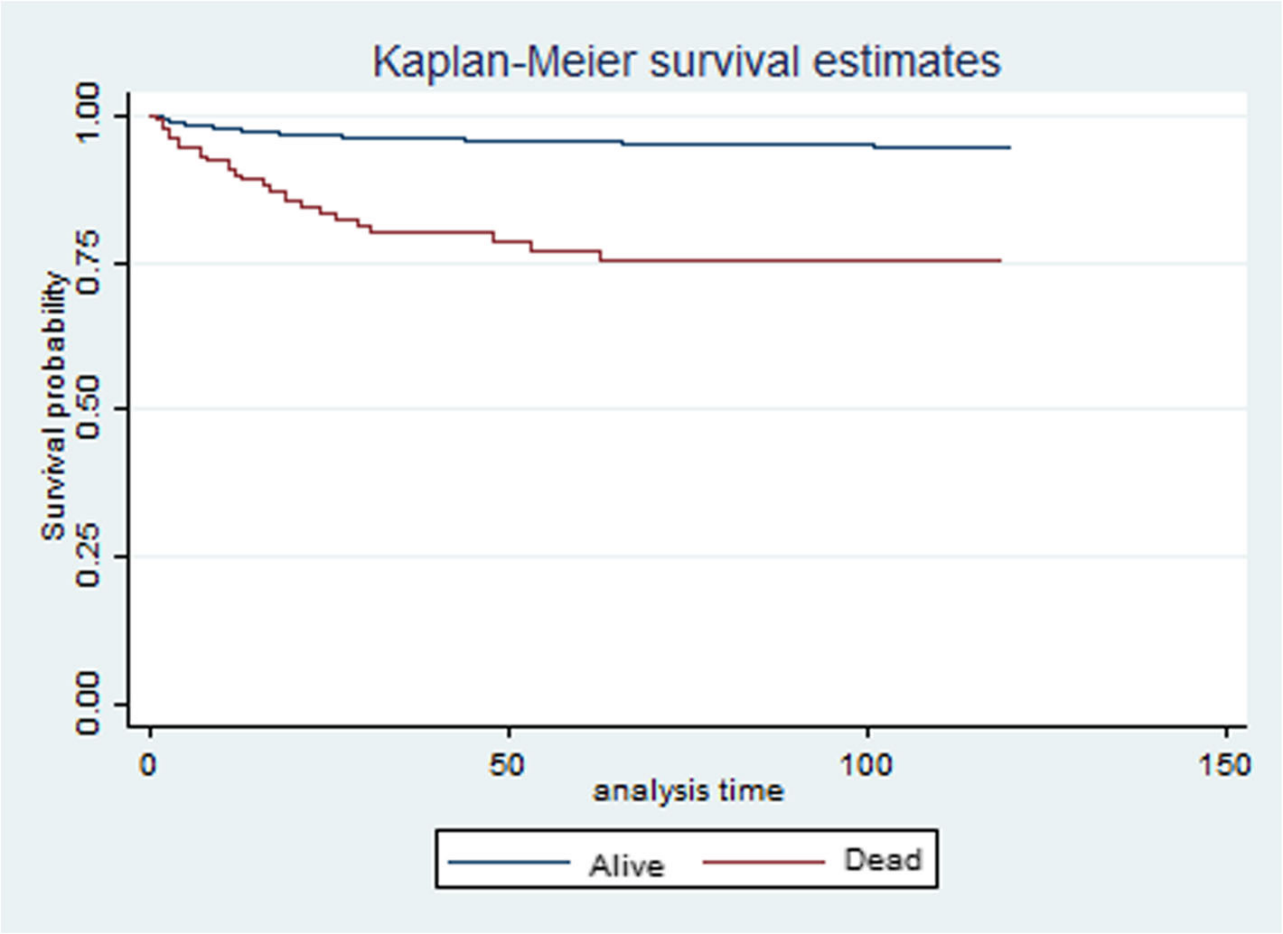
Kaplan–Meier survival probability curve by mother death status, women who die within 10 years of childbirth

The multivariate Cox model demonstrated three high risk periods (which decreased rapidly post mother’s death): less than 6 weeks after mother’s death HR = 3.45 (1.34–6.54), 6 weeks < 18 months after mother death HR =2.21(1.17–4.25) and 18 months < 3 years following her death HR = 1.58(1.03–3.19) when compared to those whose mothers were alive or died at least 1 year into the future (Table 2). The survival probabilities for each timing of mother death are represented in Fig. 2. More still, children orphaned by mother death had less survival probabilities when compared to those whose mothers were alive (*p* < 0.001) (Fig. 3).

**Table 2:** Relative risk of child death from Cox proportional hazards regression of child death, Africa Centre for Demographic Information System, South Africa 2000–2014

	Univariable			Multivariate		
	Hazards Ratio	95% CI	*p*-value	hazards Ratio	95% CI	*p*-value
Time after Mother’s Death						
< 6 weeks	8.23	4.14–12.5	< 0.001	3.45	1.34–6.54	< 0.001
6 weeks - < 18 months	6.45	3.27–9.13	< 0.001	2.21	1.17–4.25	< 0.001
18 months - < 3 years	3.18	1.98–6.59	< 0.001	1.58	1.03–3.19	< 0.001
3 years - < 8 years	0.11	0.05–8.63	0.512	0.79	0.23–6.34	0.142
≥ 8 years	1			1		
Socio economic status						
Poor	1.01	0.831–1.233	0.906	2.12	1.25–5.32	0.041
Medium	0.404	0.318–0.514	< 0.001	0.42	0.11–1.58	0.261
Unknown	0.400	0.337–0.475	< 0.001	0.22	0.02–2.67	0.345
Rich	1			1		
Age of child						
< 1 week	3.245	1.267–5.782	< 0.001	1.724	1.012–3.981	0.045
1 week - < 1 month	3.781	1.762–6.532	< 0.001	2.341	1.287–4.982	0.036
1 month - <6 months	1.246	1.010–3.529	0.045	1.642	1.121–3.762	0.023
6 months - < 1 year	1.672	1.451–3.987	0.023	1.071	0.872–3.712	0.178
1 year - < 2 years	1.213	0.672–4.218	0.136	0.876	0.291–3.751	0.122
≥ 2–10 years	1			1		
Woman’s HIV Status						
Positive	1.458	1.286–1.654	< 0.001	0.954	0.702–1.297	0.765
Negative	1			1		
Child HIV Status						
Positive	1.567	1.03–3.561	0.012	1.23	0.45–3.57	0.089
Negative	1			1		
Multiple births						
Yes	1.431	0.643–2.653	0.091			
No	1					
Father Vital Status						
Died	1.867	0.135–2.893	0.358			
Alive	1					
Child sex						
Male	1.06	0.84–1.02	0.950			
Female	1					

Cox regression model of child death on months after mother’s death, socio economic status, mother’s HIV status and sex. Unit of analysis is ‘child-month’. Explanatory variables defined at beginning of each month; child death can occur at any time within the month

Furthermore, children from the poorest households were approximately twice as likely to die compared to children from wealthier households even though poor wealth status was actually not significant in the univariate analysis. The age of the child was significantly associated with timing of mother death as shown in Table 2.The hazard risks for children aged < 1 week; 1 week < 1 month; 1 month < 6 months were 1.7; 2.3 and 1.6 respectively when compared to those aged more than 2 years. In addition, HIV positive children were at more risk as compared to those who were HIV negative even though it was not statistically significant after adjusting for all explanatory variables in the Cox regression model. Multiple births, child sex and Father’s vital status were not significantly associated with child survival in this model.The results show that mortality risk for children was very high immediately after the death of the mother as indicated by the survival probabilities in Fig. 2.

## Discussion

Our results demonstrate that the critical time of inflated mortality risk for children started immediately after their mothers’ deaths, with utmost susceptibility in the first 6 weeks of her death, decreasing substantially 18 or more months after her death. Furthermore, we found that children born from poor households were approximately twice more likely to die as compared to those from richer households. Again, the risks were more pronounced to children who were less than 6 months of age as compared to those who were 2 years and above. We also detected some ambiguity in the survival probabilities of the unknown categories of HIV Status and socio-economic status as compared to the known categories. This ambiguity could possibly be a result of the limited sample size for the unknown categories. This finding is in line with the current literature from rural South Africa by Ndirangu et al. which found that, the proportion of deaths were larger in children born to HIV positive mothers and those whose mothers’ statuses were unknown as compared to those born to HIV negative mothers in Africa Health Research Institute [7].

Our study incorporated time of woman’s death to assess its impact on child survival and woman’s HIV status. We found a very high likelihood of child death if the mother died during the postpartum period (first 6 weeks after childbirth). A significant hazard risk was also found through infancy or cessation of child breastfeeding (18 months) for children born to women who died within 18 months of childbirth. The risk remained significant for children born to women who died within 3 years of childbirth even though it was lessened. There are many possible reasons to explain the risk trends seen in this study particularly the infant and childcare within the area. Studies conducted in rural KwaZulu natal have indicated that most women gave birth at home and few deliveries are attended by trained health care workers putting which puts more women at risk of dying [22, 31]. Lack of nutrition from breastfeeding, low rates of immunization and medical health care leaves the bay more vulnerable to death or risk of infection after their mothers’ death [10, 18, 32].

Previous studies done in rural sub-Saharan Africa has reported similar findings to this study. A recent study in rural South Africa revealed that children who lost their mothers at a younger age were at higher risk of dying when compared to those whose mothers survived.Similarly, the same study indicated that children who lost their mothers when they were under 1 month were at more risk of death in comparison to older children [33]. Another previous study conducted in rural South Africa indicated that, the likelihood of a child dying enormously rose amid 2 months after their mother’s death [6]. A systematic review of studies published between January 1990 and November 2016 explored the relationship between maternal survival and child mortality. The review found that the odds of dying were more increased among motherless children [34]. Past research in sub-Saharan African countries like Ethiopia has indicated that, the risk of a child dying is immense less than 6 weeks after their parent’s demise [5, 10, 17, 19]. Most of these studies in rural sub-Saharan Africa have indicated that most child deaths occur in the neonatal period after the mothers’ deaths. This is largely to lack of prenatal and postpartum care in the growth of newborns, with undernutrition contributing more than 40% of child deaths [35]. The results of the study remained significant even after the inclusion of other potential confounders associated with mother and child death (wealth status, age of the child, multiple births, father’s vital status, sex of child, mother and child’s HIV status). It is important to highlight also that the study found that children born from poor households and those who were younger than 6 months after their mothers’ deaths had a high risk of dying.

There are quite many complementary reasons for the possible explanations of our results, with nutrition and caregiving the likely major factors. Any death of a mother will likely lead to the death of the child, particularly those children who are completely reliant on their primary caregivers [36]. Research have identified malnutrition as one of the critical causes of poor health and child deaths, particularly in communicable diseases [6].Usually after the death of the mother, high risk of child deaths could be a result of inadequate feeding mechanisms from the next of kin in already resource depleted environment [37]. One of the critical implications of HIV/AIDS among the adults in poor rural households is poverty [38]. Prolonged adult ailment and later death may lead to loss of a breadwinner and reduced care to children. As result, there is an increased likelihood of lack of support and care, which in turn directly reduce the survival chances of the child.More robust interventions from the government are required in rural setups where HIV/AIDS pandemic is still high [2, 28].

Lack of proper communal backing ways to help households who take care of orphaned children,

creates possible additional vulnerabilities for orphaned children. Also, the death of a woman impacts negatively on her partner and/or other relatives now responsible for childcare and their capacity to cope whilst grieving. This finding is supported by a study conducted in Butajira (Ethiopia) which showed lack of support from fathers after the death of the mother as well as the struggle and challenges faced by extended families in playing the role of caregivers [39].

The major strength of this study is the usage of a continual longitudinal data for decisive events over a protracted period and follow-up, to investigate the survival paths of children who die because of maternal mortality. The longitudinal nature of the datasets permits an attentive monitoring of both deaths and births in the study area. Nonetheless, there are some data limitations like the rarity of multiple births and maternal deaths even in areas like Africa Health Research Institute were mortality is generally high. Secondly, there is a high possibility of the same factors influencing mortality in both children and their mothers which makes it difficult to take a conclusive stance on the causality relationship between maternal and child deaths. Although we adjusted our analyses for socioeconomic asset score of the household, mother’s and child’s HIV status, age of child, multiple births, father’s vital status and sex of the child, there is still a possibility of residual confounding between the predictor variables.

This study has found that time of woman’s death plays a critical role on child survival and there are many factors leading to those deaths. There are many effective interventions that could be done to prevent the child deaths after their mothers’ deaths. These include expansion of educational programmes and women support groups aimed at improving health systems in rural areas that can adequately tackle newborn, child and maternal health challenges. Also, governmental policies aimed at addressing the inequitable distribution of resources and money should be prioritized. Thus pairing clinical care with community-based interventions and policies to tackle challenging maternal death risk factors, incorporating educational and awareness programs are very critical for child survival. Some of the interventions and policies include accessibility of antiretroviral therapy and information on prevention of mother to child transmission and usage of contraceptives precisely in high HIV/AIDS burdened areas [40–42].

## Conclusion

These results show women’s death is influential upon the risk and timing of child death, and the association between child and maternal mortality deserves more scrutiny by research investigators and policy guiders. The imminent consequence of the death of a mother is the end of her role to her family and household as a breadwinner through income earning as well as protecting and envisaging education to children. The findings of this study highlight the need to develop significant policy information on the implications of maternal death on child survival especially in rural sub-Saharan Africa where HIV/AIDS prevalence is still high.

## Abbreviations

AHRI: Africa Health Research Institute
AIDS: Acquired Immune Deficiency Syndrome
HIV: Human immune Virus
HR: Hazard Ratio
MEPI: Medical Education Partnership Initiative
TB: Tuberculosis
